# Supplementary material for: A new perspective on the evolution of “Kawara” roof tiles in Ryukyu: A multidisciplinary non-destructive analysis of roof tile transition at Shuri Castle, Ryukyu Islands, Japan
Source: PLoS One. 2022 Nov 16;17(11):e0277560. doi: 10.1371/journal.pone.0277560 (PMC9668144; doi:10.1371/journal.pone.0277560)
Supplement: S2 Appendix — (PDF) [file pone.0277560.s002.pdf]

Supplementary Information

A new perspective on the evolution of "Kawara" roof tiles in Ryukyu: a multidisciplinary non-destructive analysis of roof tile transition at Shuri Castle, Ryukyu Islands, Japan.

Hiroaki Aoyama*†[1], Kaishi Yamagiwa*†[2,3,4], Wataru Taira*[5], Takeshi Kon[1]

[1] Research Planning Office, University of the Ryukyus, Nishihara, Okinawa 903-0213, Japan.

[2] Research fellow of the Japanese Society for the Promotion of Science, Chiyoda, Tokyo 102-0083, Japan.

[3] Research Institute for Islands and Sustainability, University of the Ryukyus, Nishihara, Okinawa 903-0213, Japan.

[4] Center for Strategic Research Project, University of the Ryukyus, Nishihara, Okinawa 903-0213, Japan.

[5] Center for Research Advancement and Collaboration, University of the Ryukyus, Nishihara, Okinawa 903-0213, Japan.

*Contributed equally

†Corresponding authors: Kaishi Yamagiwa (yamagiwa@lab.u-ryukyu.ac.jp), Hiroaki Aoyama (aoyama@lab.u-ryukyu.ac.jp)

Supplementary Methods

Fig. S1-S10

Supplementary Methods

CT data wrangling (Supplementary methods)

Data wrangling on the pore data was performed using the software package NYSOL[37] version 2.4.2 (NYSOL Corporation, Osaka, Japan). First, to standardize the pore volume detection limit among samples, pores smaller than one voxel volume of the lowest resolution sample (0.0018 mm^3 in MCO018, MCO019, and MCO020) were removed from all samples. This process removed approximately 28% of all pores. Next, pores with low defect probability values were removed because they were likely to be noise. The threshold value changed depending on the pore volume. For small pores of less than 1 mm^3, the threshold value was 1.0 (the default value on the software), and pores with a smaller value were removed. Large pores ($\geq$ 1 mm^3) were less likely to be noise and tended to have smaller probability values; therefore, all data were used regardless of defect probability values. During this process, approximately 88% of the pores were removed.

Calculations of eight values for internal microstructural features (Supplementary methods)

The eight values for the internal microstructural features were calculated as follows (calculations were performed using Microsoft Excel):

(1) Density-S: weight of sample/sample volume (i.e., total pore volume + material paste volume).

(2) Density-M: weight of the sample/material paste volume.

(3) Median-vol: median pore volume (searched on NYSOL).

(4) Mode-vol: mode of pore volume (searched on NYSOL).

(5) Dispersion-vol: dispersion of pore volume (calculated using NYSOL).

(6) RLV: total volume of large ($\geq$ 1 mm^3) pores / total volume of all pores.

(7) Num-density: number of pores (n) / sample volume (cm^3).

(8) RLN: number of large pores ($\geq$ 1 mm^3) / number of all pores.

A

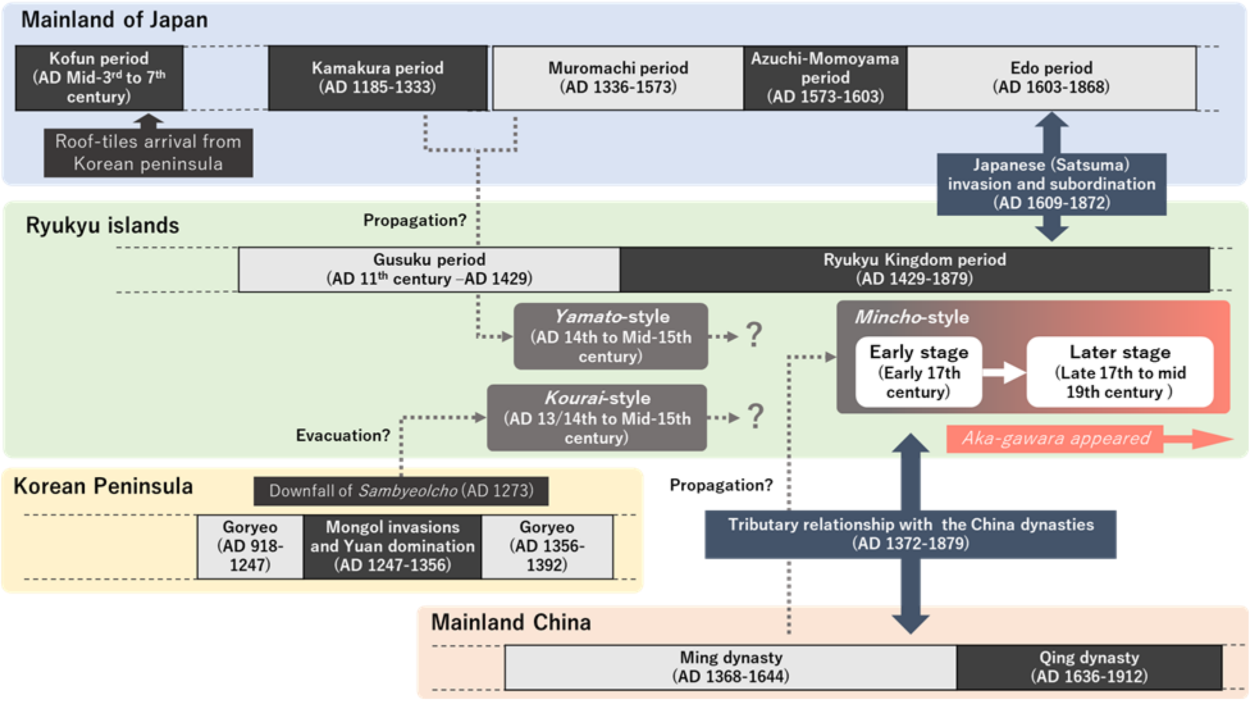

B

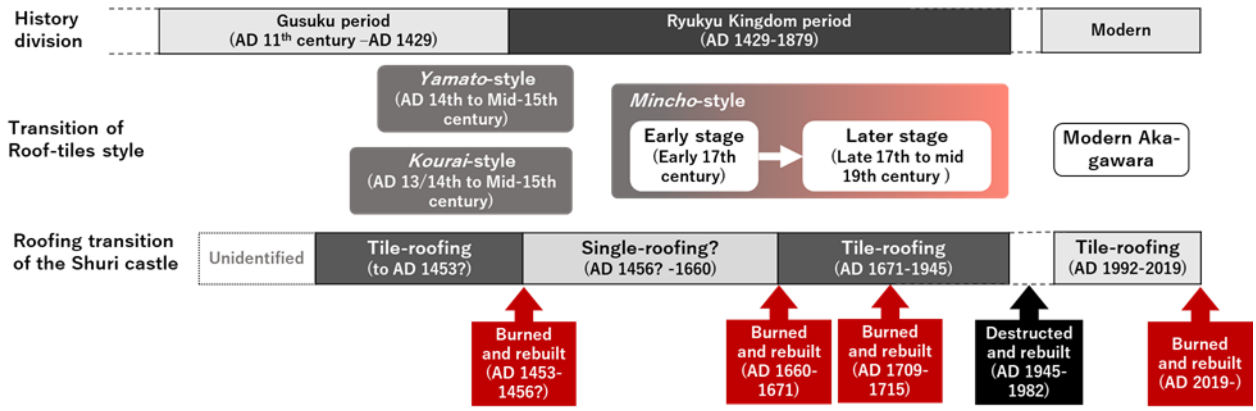

S1 Fig. Chronology of the Ryukyu Kingdom and Shuri Castle.

(A) Diagram of relations with neighboring countries regarding roof tiles. (B) Diagram of Shuri Castle and roof tiles.

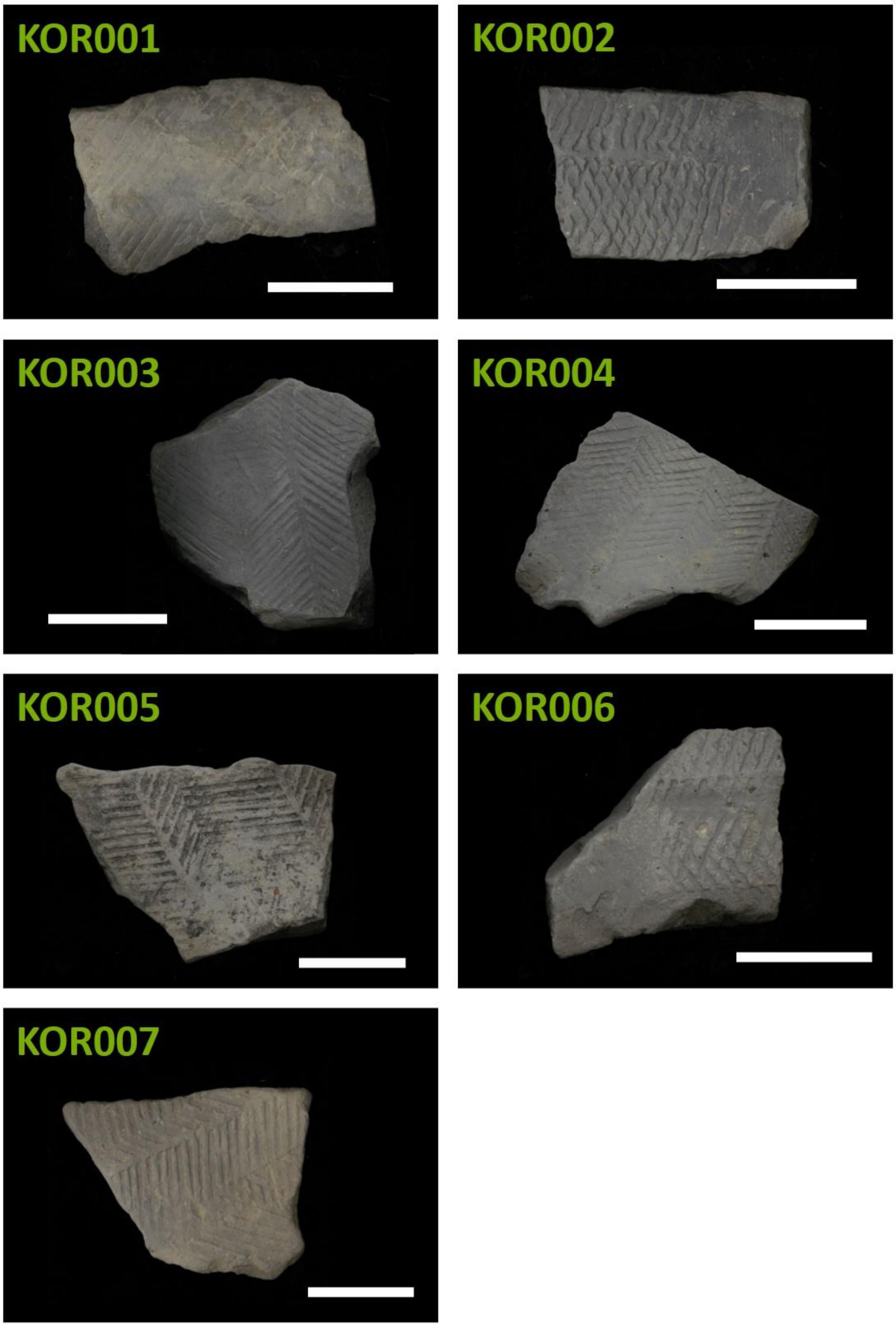

S2 Fig. Images of Kourai samples.

The images were captured using a bizhub C368 multifunction printer (Konica Minolta, Japan). Each white scale bar represents 4.5 cm. These images show the characteristic surface and do not necessarily match the images used in the color analysis.

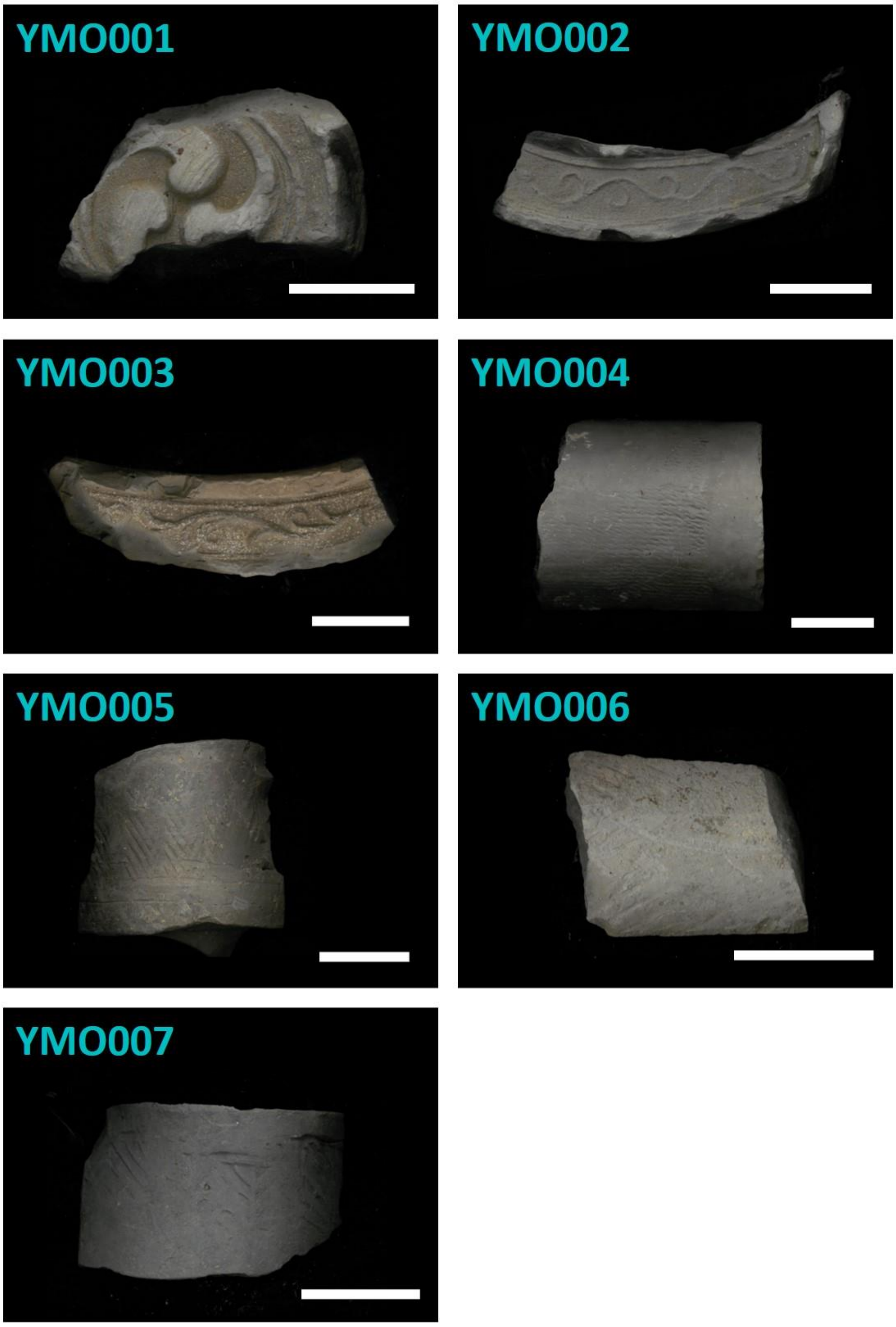

S3 Fig. Images of Yamato samples.

The images were captured using a bizhub C368 multifunction printer (Konica Minolta, Japan). Each white scale bar represents 4.5 cm. These images show the characteristic surface and do not necessarily match the images used in the color analysis.

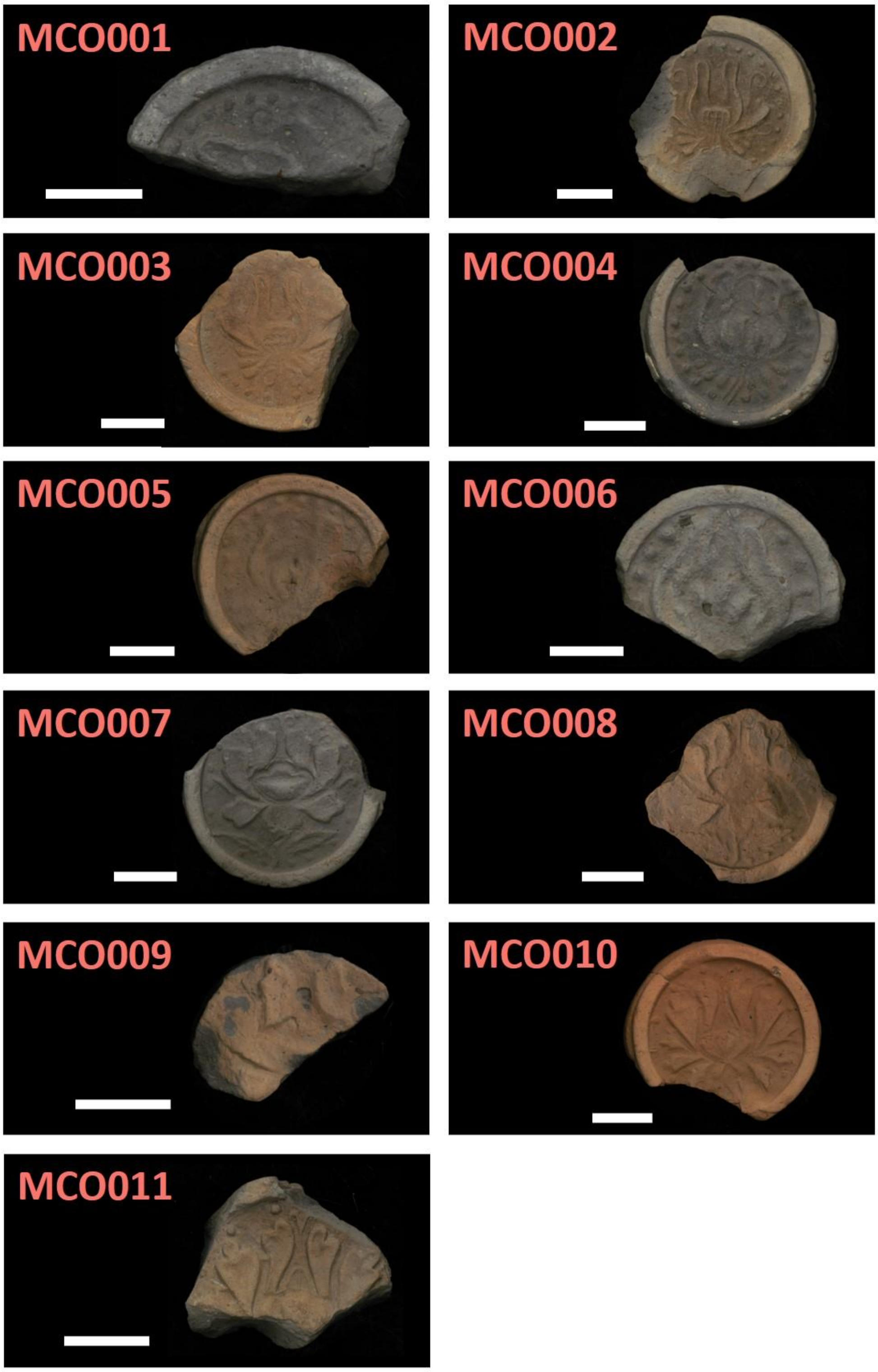

S4 Fig. Images of Minchō (initial phase) samples.

The images were captured using a bizhub C368 multifunction printer (Konica Minolta, Japan). Each white scale bar represents 4.5 cm. These images show the characteristic surface and do not necessarily match the images used in the color analysis.

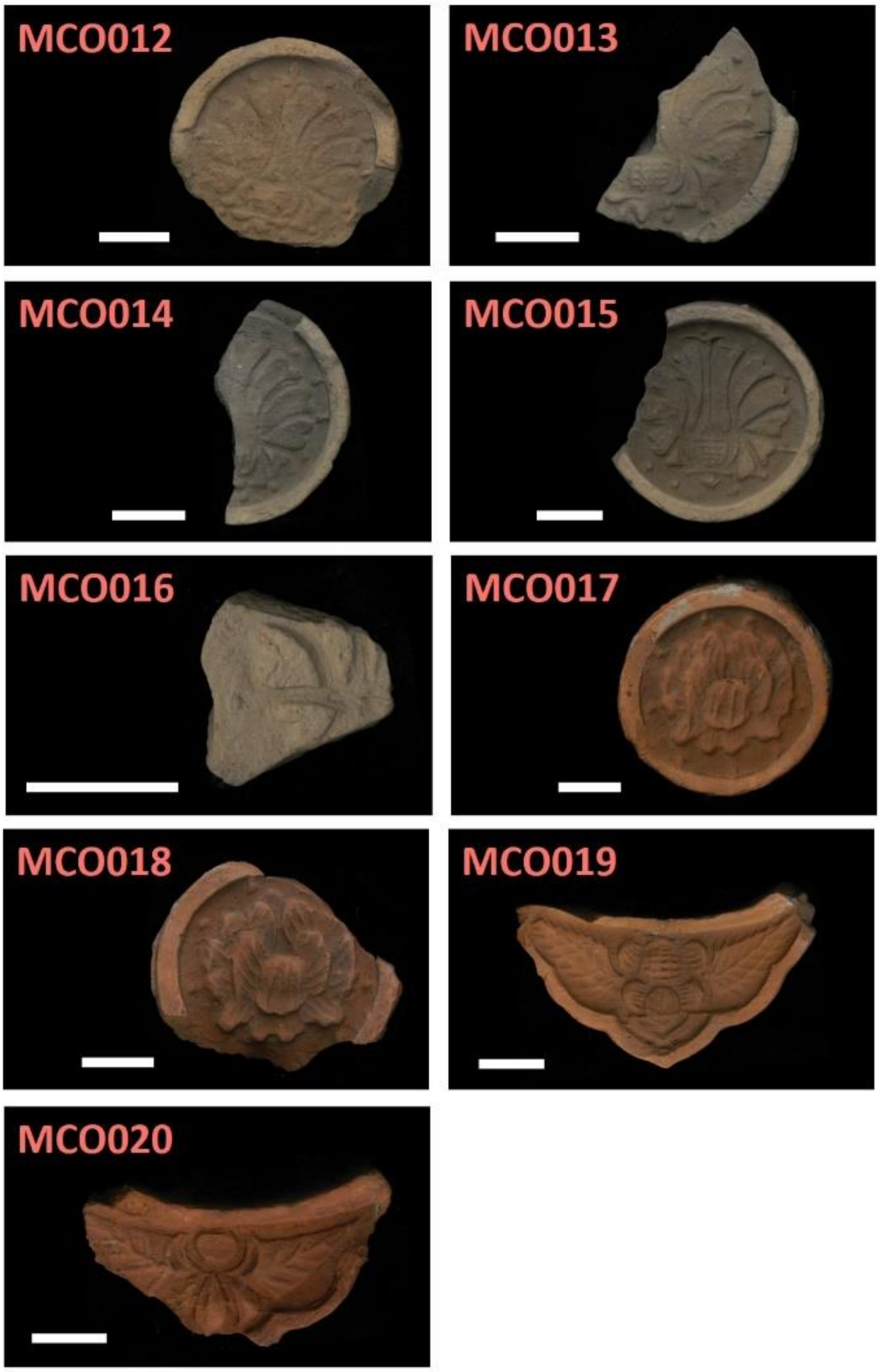

S5 Fig. Images of Minchō (late phase) samples.

The images were captured using a bizhub C368 multifunction printer (Konica Minolta, Japan). Each white scale bar represents 4.5 cm. These images show the characteristic surface and do not necessarily match the images used in the color analysis.

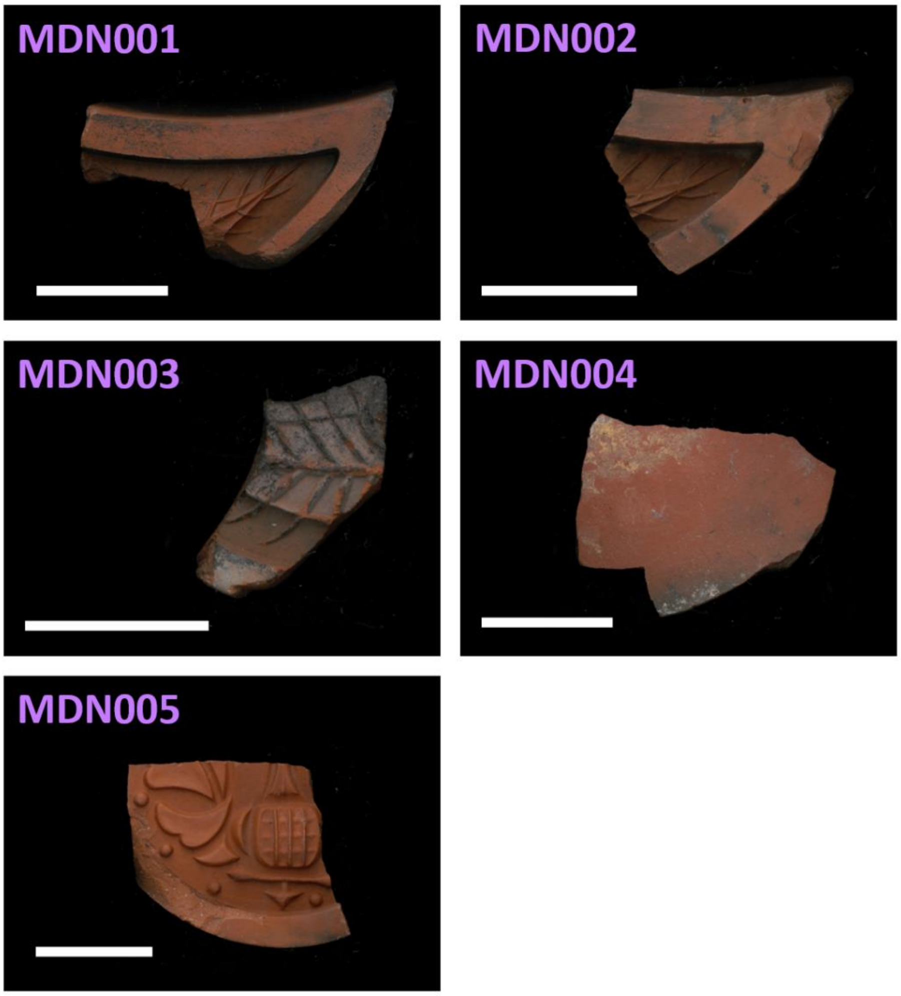

S6 Fig. Images of Modern samples.

The images were captured using a bizhub C368 multifunction printer (Konica Minolta, Japan). Each white scale bar represents 4.5 cm. These images show the characteristic surface and do not necessarily match the images used in the color analysis.

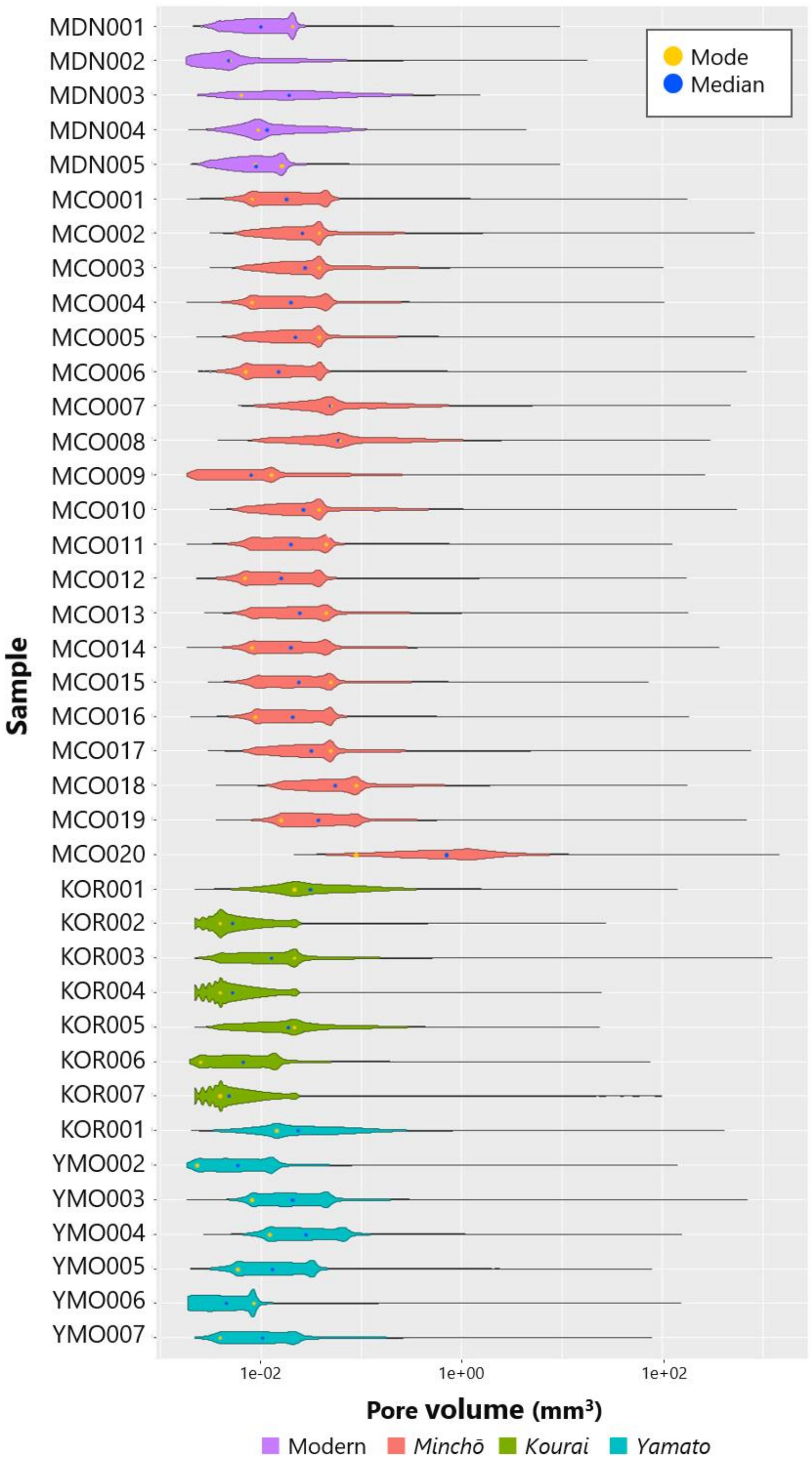

S7 Fig. Violin plot of the pore volume.

Each violin plot shows the distribution of the pore volume and mode as a yellow plot and the median as a blue plot. Violin plots were plotted using ggplot2 in R.

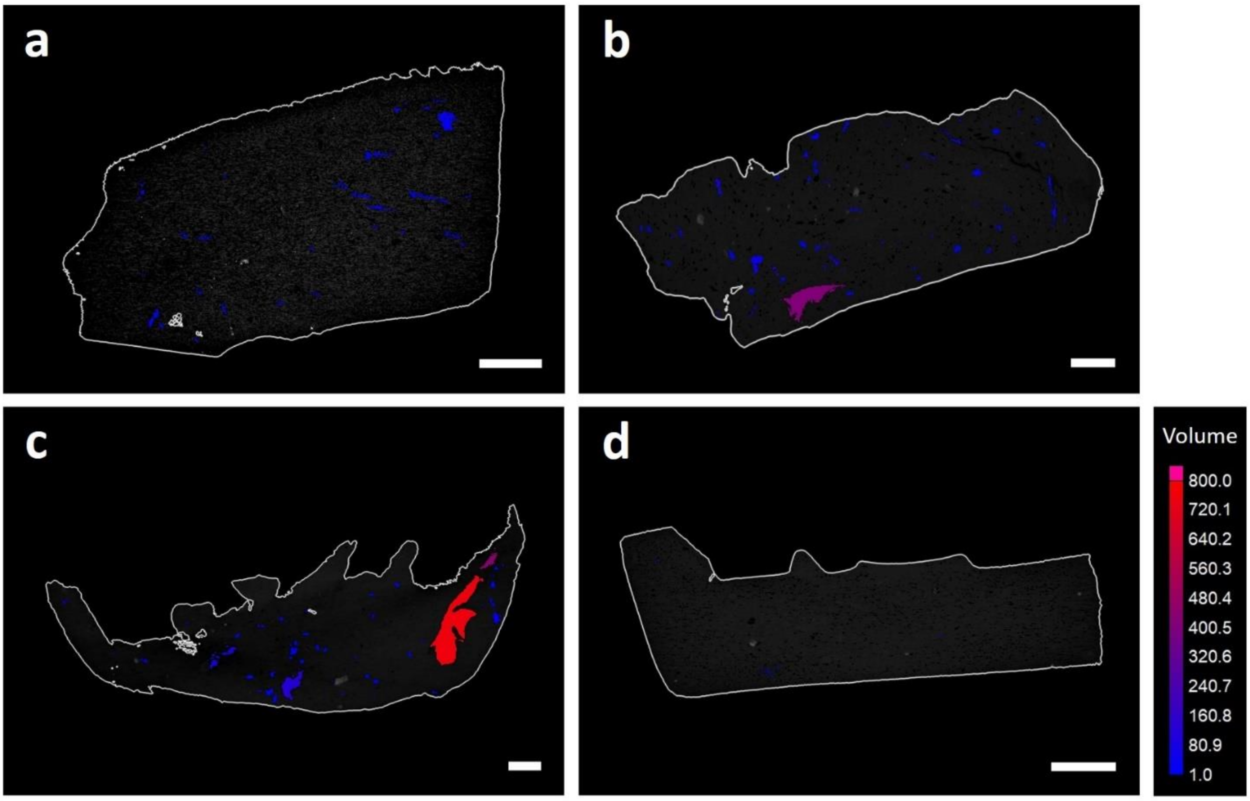

S8 Fig. Examples of pore analysis.

Pores larger than 1.0 mm^3 are shown in a scale from blue to red on the CT tomograms. The smallest pores (< 1.0 mm^3) are not shown here. The gray spots show the difference in material density, not pores. However, the scale of the density difference is not uniform in these images and is not meaningful information. Each white scale bar represents 1 cm. (a) Kourai style (KOR004) (b) Yamato style (YMO001) (c) Minchō style (MCO017) (d) Modern tiles (MDN005).

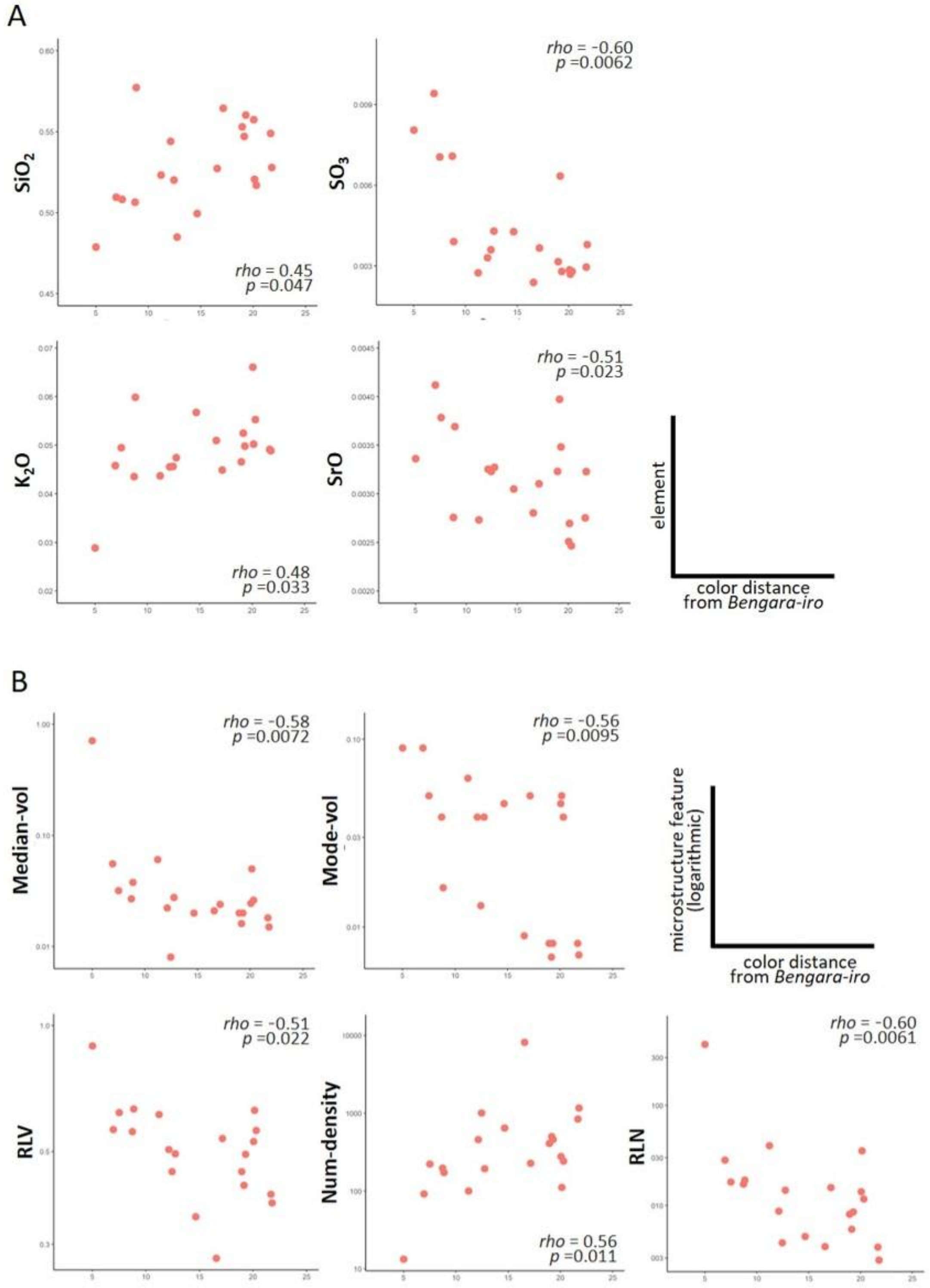

S9 Fig. Correlation analysis of the color distance from Bengara-iro with the roof tile features.

The Spearman's correlation coefficient (rho) and its associated p-values are shown. The scatter plots are shown only for features that had significant correlations. For the other features, see S8 and S9 Tables. (A) Elements (SiO2, SO3, K2O, and SrO). (B) Microstructural features (Median-vol, Mode-vol, RLV, Num-density, and RLN).

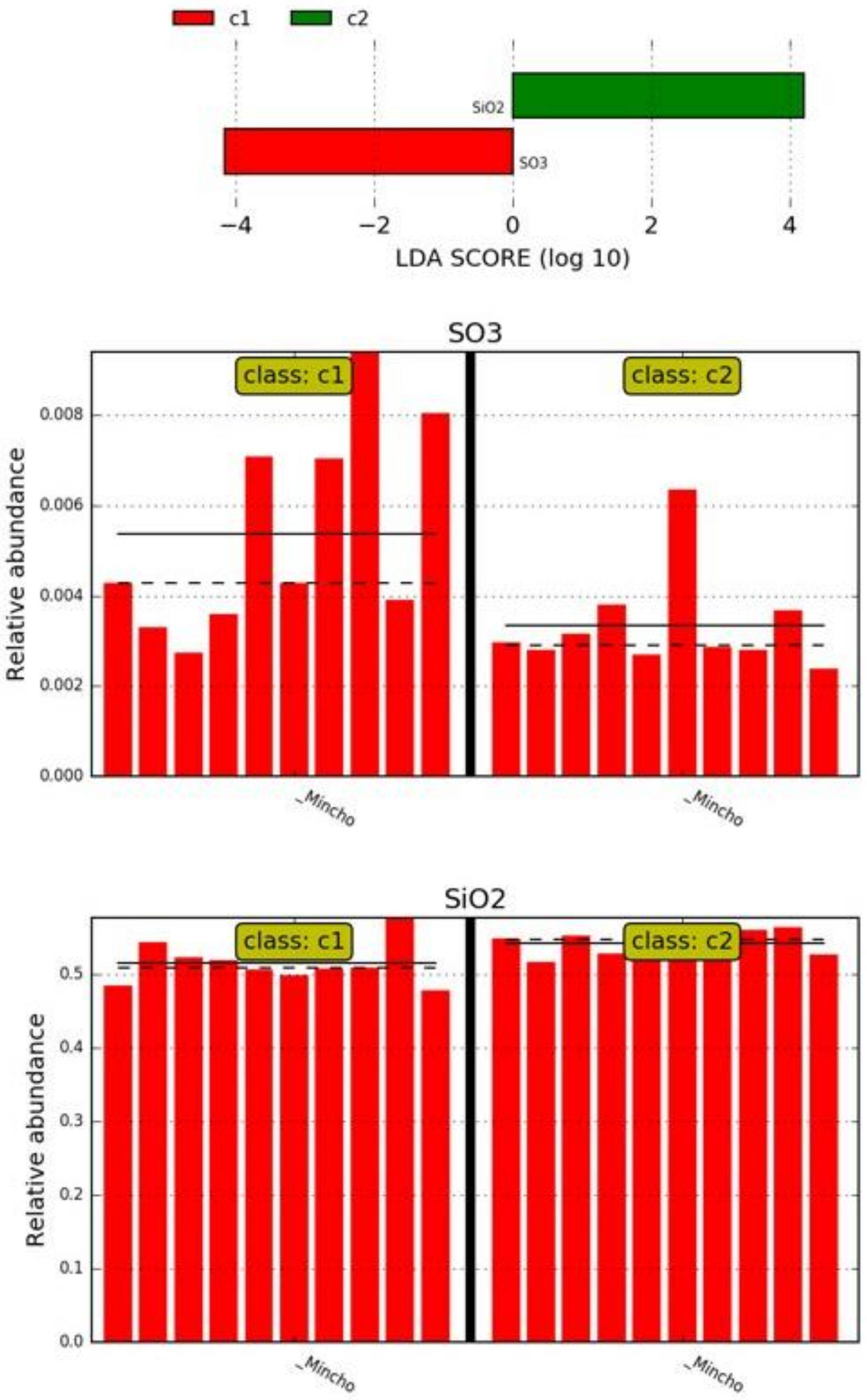

S10 Fig. Linear Discriminant Analysis Effect Size (LEfSe) of the chemical components in Minchō-style roof tiles.

LEfSe revealed a list of features that enabled discrimination between the color clusters in the Minchō-style roof tiles. The horizontal bar graph shows the LDA scores of the reddish and gray clusters. The histograms show the presence of three featured elements in the Minchō-style roof tiles. The horizontal straight lines in the panel indicate the group means, the dotted lines the group medians.
